# Social Support, Metabolic Syndrome, and White Matter Hyperintensities in Mexican Americans

**DOI:** 10.1093/geroni/igaf122.2466

**Published:** 2025-12-31

**Authors:** Zoraida Garcia, Jordana Breton, Lourdes Romañach-Álvarez, Elizabeth Muñoz

**Affiliations:** University of Texas at Austin, Austin, Texas, United States; The University of Texas at Austin, Austin, Texas, United States; The University of Texas at Austin, Austin, Texas, United States; University of Texas at Austin, Austin, Texas, United States

## Abstract

Mexican Americans (MA) are disproportionally affected by poor cardiovascular health, making them susceptible to brain impairment through White Matter Hyperintensities (WMH), increasing dementia risk. Metabolic Syndrome (MetS) is an early marker of cardiovascular disease, and one potential sociocultural factor that may be protective against conditions of MetS among MA is social support. We examined the link between MetS and WMH and whether social support moderated this association. Our sample consisted of 1,050 MA adults (ages 50-91) from the Health and Aging Brain Study: Health Disparities (HABS-HD). MetS was diagnosed using the Adult Treatment Panel III criteria (MetS+/MetS-), WMH volumes were measured through a 3T MRI System with higher values indicating more damage, and social support was calculated with the 12-item Interpersonal Support Evaluation List. We used linear regression to examine the independent effects of MetS and WMH volume, and an ANCOVA model to assess the interactive effects of social support on MetS and WMH volume. Models adjusted for age, sex, and education. Independent effects revealed that MetS+ status was significantly associated with higher WMH volumes (B = 0.32, p< 0.05, R2=0.25). This association did not vary by levels of social support WMH (F[5,1041]=2.91, p=0.08, ηp2<.001). Results suggest that MetS+ exacerbates WMH, and that social support does not impact the link between MetS and WMH. Future studies should investigate other potential sociocultural resiliency factors that may buffer the impact of MetS on WMH in middle-aged and older MA adults.

